# Adiponectin and HbA1c levels among Indian patients with diabetes mellitus

**DOI:** 10.6026/973206300200202

**Published:** 2024-02-29

**Authors:** Mohammad Arif, Shreya Nigoskar, Manish Kumar Verma

**Affiliations:** 1 Department of Biochemistry, Index Medical College & Research Center Indore, Madhya Pradesh, India; 2 Department of Biochemistry, Rajashri Dashrath Autonomous State Medical College Ayodhya, U.P, India

**Keywords:** Adiponectin, Glycosylated Haemoglobin, Type 2 Diabetes Mellitus, Metabolic Disorder, Inflammatory Biomarkers

## Abstract

Adiponectin is closely related to glucose metabolism and newly diagnosed type 2 diabetes mellitus (T2DM), and other kinds of diabetes linked to the risk of T2DM. Therefore, it is of interest to report the correlation between adiponectin levels and glycosylated hemoglobin (HbA1c) as a diagnostic marker of T2DM and healthy control. Total 210 participants were included of IPD & OPD healthy controls with glycosylated hemoglobin levels under 6% were included. Blood samples, collected using sterile clot activator or plain vials, were stored at -20°C. The biomarker score that comprised significant differences in age, gender distribution, and metabolic indicators are seen between the diabetes (n=105) and control (n=105) groups. Increase in both Adiponectin and HbA1c% Mean±SD (6.86±0.23, p<0.0001; 22.71±2.01; p<0.0001) is indicative of deteriorating glycaemic control and an accompanying rise in inflammatory response. Positively correlate adiponectin levels with HbA1c levels (r2=0.398; p<0.0001), suggesting a link between inflammatory response and glucose control. Lower adiponectin levels are statistically associated with diabetes. Diabetes and adiponectin were negatively correlated and positive linear relationship between HbA1c and adiponectin levels. Adiponectin may be a significant factor useful in understanding the pathophysiology; they are likely to be straight forward instruments for predicting future risk of diabetes.

## Background:

Diabetes mellitus is a group of metabolic diseases characterized by hyperglycemia resulting from defects in insulin secretion and insulin action or both. Chronic hyperglycemia is linked to long-term damage, abnormalities, and loss of normal function in several organs, including the heart, blood vessels, kidneys, eyes, and nerves [1]. Type 2 diabetes is frequently accompanied with obesity (T2D). At least 90% of patients diagnosed with type 2 diabetes globally are believed to be fat or overweight [2]. Obesity is one of the main risk factors in the onset of type 2 diabetes. Peripheral tissues experiencing insulin resistance are linked to the rise in lipid content in the nucleus of adipocytes, myocytes, and hepatocytes that occurs concurrently with obesity [3]. Furthermore, several popular T2D drugs (such as insulin, thiazolidinediones, and derivatives of sulphonyl urea) increase body mass, which exacerbates insulin resistance. Type 2 diabetics who also have concurrent obesity have a much higher risk of several co-occurring conditions, especially those involving the cardiovascular system and chronic renal conditions [4]. One of the main players in the inter-relationship between obesity and insulin resistance is adiponectin, an insulin sensitizer produced from adipose tissue that also happens to be a significant risk factor for type 2 diabetes [5].

The processes that link lipid metabolism with the progression of type 2 diabetes, impaired glucose tolerance and insulin resistance is of concern. It seems that the changed actions of adipose tissue can account for, at least in part, of the higher Potential for type 2 diabetes among people who are overweight [6]. Metabolic syndrome (MetS), a cluster of conditions including obesity, hyperglycemia, dyslipidemia, and hypertension, occurs around five years prior to the onset of diabetes and is associated with an increased risk of developing the disease. In people with diabetes, common diabetes risk factors such as obesity, hypertension, and dyslipidemia frequently coexist [7]. Consequently, newly predictable elements are required to stop various disease processes leading to complications from diabetes [8]. Adipokines could function both locally in adipose tissue and systemically in circulation via secretion [9]. Adipokine levels have been shown to be different in patients having renal impairment in earlier research. Furthermore, research has demonstrated that specific adipokine types regulate dysfunction of endothelial cells, oxidative stress, and inflammatory processes, which in turn may cause kidney damage [10].

Adiponectin and leptin are two adipokines whose roles in diabetes complications have been extensively researched. Nevertheless, there is uncertainty regarding the relationships between several adipokines, mostly released by white adipose cells, and diabetes problems [11]. Adiponectin is a well-known adipokine, it was not included in this investigation since its level in plasma has previously been linked to diabetic nephropathy [12. Frequent evaluation of circulating adipocytokines is also helpful in determining the optimal course of action in relation to these crucial therapeutic indicators. Therefore, it is of interest to determine if circulating levels of glycosylated hemoglobin and adiponectin show any discernible relationship with T2DM in this glycosylated hemoglobin.

## Methods:

## Research design:

Total 210 participants study was conducted in the Department of Biochemistry; include, who visited the Outpatient & Indoor patient Department of Medicine, Index Medical College, Hospital & Research Center, Indore, India. We included in the study. Study conducted from November 2021 to September 2023 and consider group of type 2 diabetes mellitus patients & Control group. Ethical clearance was taken by Institutional Ethic Committee. Healthy controls were recruited from the hospital who visited the outpatient Department for screening glycosylated hemoglobin level but it was found less than 6%. Disposable syringe was used in a sterile clot activator or plain vial for all aseptic precautions of venous. Even, the E.D.T.A vial was selected by those patients who were fasted at least 10-12 hours as well as the sample was stored at -20°C.

## Inclusion criteria:

[1] Patients withi type 2 Diabetes Mellitus (according to ADA criteria for diabetes)

[2] Pre-diabetes (Impaired Fasting Glucose/Impaired Postprandial Glucose)

[3] Age group > 30

## Exclusion criteria:

[1] Patients withi any systemic disease e.g. asthma, chronic obstructive pulmonary disease (COPD), malignancies, Sexually Transmitted Diseases, cardiovascular disease

[2] Patients withi Type 1 diabetes mellitus.

## Evaluation of biomarkers:

High performance liquid chromatography (HPLC) based HbA1c autoanalyzer Bio-Rad D-10 (Bio-Rad Laboratories, USA), Adiponectin by Sandwich ELISA Method.

## Statistical analysis:

The study has used SPSS 27 for effective analysis. The authors have expressed the continuous data as Mean±SD deviation while the discrete data were expressed as frequency and its respective percentage. The Pearson's correlation for analyzing the association of glycosylated hemoglobin level with adiponectin level is shown. The graphs were plotted in MS Excel and the equation of the trendline was generated. The significance level of was P<0.05.

## Results:

**Figure 1:**
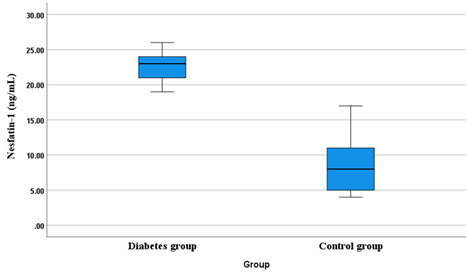
Boxplots showing the adiponectin level in each group

Table 1 compares two groups based on their initial conditions: those with diabetes (n=105) and those without (Control, n=105) Figure 1 . Mean age is 52.03 years for the Diabetes group and 39.13 years for the Control group. The Diabetes group is significantly more male (71.42%) than the Control group (47.04%). The Diabetic group's glycosylated hemoglobin level is greater than the Control group's glycosylated hemoglobin level (5.57%). The levels of the metabolic hormone Adiponectin (22.71 ng/ml) in the Diabetes group are substantially higher than those in the Control group (8.36 ng/ml). Differences in age distribution, gender composition, and metabolic indicators are just a few of the ways in which diabetes sets the two groups apart.

The IQR for the diabetes group (1.275 ng/ml) is narrower than the IQR for the control group (1.045 ng/ml). This indicates less variability in Adiponectin levels among individuals with diabetes compared to those without diabetes. To account for differences in central tendency, calculate the CV for each group (IQR divided by the median and expressed as a percentage). A lower CV for the diabetes group would further substantiate the observation of less variability in Adiponectin levels. Figure 3 below shows the boxplot diagram of Adiponectin Level for each group.

**Figure 2:**
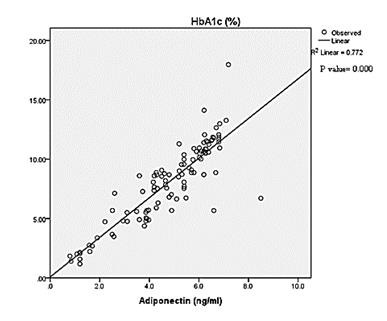
Scatter diagram showing the distribution of HbA1c and adiponectin level in control group

**Figure 3:**
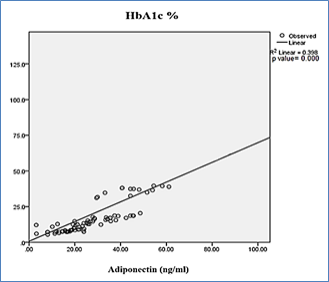
Scatter diagram showing the distribution of HbA1c and adiponectin level in case group.

Figure 2 depicts the significant positive correlation was found in control between the HbA1c (%) and Adiponectin (ng/ml) risk factors. Obtained data was significantly positive at p<0.000 level. Reproducible with strong accuracy was observed as predicted from higher correlation coefficient r2=0.772 value. Figure 3: showed the case relationship between the HbA1c (%) and Adiponectin (ng/ml) risk factor. As depicted from above figure. Positive correlation exists between the HbA1c (%) and Adiponectin (ng/ml) p value significant p<0.000 level. The presented data was significant differences between the risk factors were identified, but less reproducible data was produced and good predicted statistical analysis was shown here in Figure 3. As predicted by correlation coefficient r2=0.398 value.

**Figure 4:**
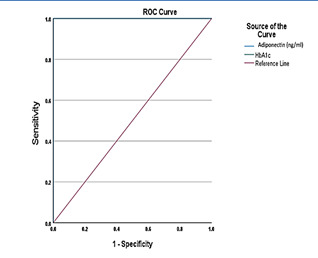
ROC analysis for considering adiponectin and hba1c in type 2 diabetes mellitus

Figure 4 shows the coordinates of ROC for each variable (Adiponectin and HbA1c). For Adiponectin, the author suggests cut-off for Adiponectin can be chosen (with sensitivity at 1 and 1-specificity at 0). Table 2 shows the performance of Adiponectin and HbA1c of Type 2 Diabetes Mellitus. The results showed that the area under ROC curve (AUC) predicted by Adiponectin and HbA1c was 0.943 (95%CI: 0.942-0.998) and 0.910 (95%CI: 0.929-0.994) the difference was considered statistically significant (p < 0.0001); which represented a high diagnostic accuracy. When Adiponectin cut off value 0.692, the maximum Adiponectin and HbA1c Youden index was 0.189 & 0.161, and the corresponding sensitivity and specificity of diagnosing Adiponectin (µg/ml) were 98.1% and 96.8%, respectively. In univariate analysis (Table 3) was correlation coefficient Adiponectin and HbA1c of Type 2 Diabetes Mellitus, Adipokines (µg/ml) negative correlation & highly significantly was showed with HbA1c, at r= 0.675** ; p<0.000 level respectively and age correlated with Adipokines (µg/ml) r= -.077 p>0.267 negative correlation & not significantly respectively; age correlated with HbA1C (%) r= -.115 p>0.098 negative correlation & not significantly respectively; suggesting that, on average, as higher Adiponectin levels is associated with increasing age.

## Discussion:

Adipokines may have a crucial part in the development of diabetes mellitus, according to some recent studies. Adipokines may not possess a clinically noteworthy effect on the long-term prognosis of individuals with diabetes and chronic renal disease. Finding a predicted factor among individuals with long-term diabetes problems was the aim of this investigation. Patients with renal insufficiency had considerably higher levels of plasma adiponectin and glypican-4 [13]. These adipokines revealed a positive correlation to urine albumin excretion. It is also found that there is positive correlation to estimated glomerular filtration rate. Renal progression's relative risk increased with rising adiponectin levels towards dialysis rose as well, although in an independent manner. Visfatin and glycopan-4 did not predict the development of any macrovascular or microvascular problems. The complication of diabetic nephropathy and cerebrovascular sequelae was elevated by glucose fluctuation. Glypican-4 and adiponectin were linked to renal function and may be able to forecast the course of renal disease. Studies predicted significant risks for both diabetic nephropathy and cerebrovascular consequences, such as glucose fluctuation [14].

It has recently been discovered that the adipokine adiponectin serves as a biomarker for systemic inflammation. Although cardiovascular disorders are caused by atherosclerosis, it is unclear if Adiponectin speeds up this process [15]. In the current investigation, possible relationship between systemic inflammation and diabetic nephropathy has been investigated and changes in plasma Adiponectin levels. Furthermore, we looked at how Adiponectin functions physiologically in vascular smooth muscle cells (VSMCs). All things considered, these results imply that by stimulating Adiponectin production, VSMCs' MAPK pathway may facilitate MCP-1 activation in a diabetic environment. may potentially hasten atherosclerosis [16].

Insulin resistance and dysfunction are the two main disorders that decrease the body's ability to regulate blood glucose levels and contribute to the pathogenesis in type 2 diabetes mellitus. Adipose tissue secretes the hormone adiponectin, which has insulin-sensitizing qualities and participates in glucose metabolism. Insulin resistance is caused by reduced reduction in the skeletal muscle cells' uptake of glucose, fatty acid oxidation, and rise in free fatty acid levels when adiponectin levels are low [17]. Another adipokine made Adiponectin is released by fat tissue and operates with the hypothalamus to reduce appetite and increase energy expenditure, which in turn regulates how much food is consumed. In the pathogenesis of type 2 diabetes mellitus, Adiponectin is essential. The goal of the study was to look at the relationship between Sudanese individuals who have blood adipokine levels in those with type 2 diabetes in relation to glycemic management and metabolic dyslipidemia. Individuals who had type 2 diabetes mellitus enjoyed elevated blood levels of Adiponectin and lower levels of adiponectin. Adiponectin or Adiponectin levels were shown to be significantly correlated with both metabolic dyslipidemia and glycemic control [18]. A study was conducted to investigate the connection between specific lipid indices and lipid ratios in type 2 diabetic individuals who have glycosylated haemoglobin (HbA1c). The LDL-C/HDL-C ratio can help determine and reduce the risk of cardiovascular disease in people with type 2 diabetes because of impaired lipid metabolism [19].

Data from the participants was examined according to various parameters. In comparison to the men, the Women's values were much higher for triglycerides (TGs), cholesterol levels that were high-density lipoprotein (HDL-C) and LDL-C values (low-density lipoprotein cholesterol) (p<0.001), BMI (p=0.002), and HbA1c (p=0.009) [20]. The HbA1c levels of the research participants were used to categorize them into two groups: good glycemic index <7% and poor glycemic index >7%. All the parameters were similar in both groups, except for TGs and HbA1c. There was no significant association found with the other variables in the correlation study of HbA1c and other variables, despite a noteworthy correlation with TG. According to the results of the linear regression, HbA1c values were linked to TGs and did not depend on FPG levels, age, BMI, a TC, LDL-C, HDL-C, or BMI [21].

Patients who suffer from dyslipidemia- a condition that raises the risk of cardiovascular diseases (CVDs) are more likely to have type 2 diabetes (T2DM). To determine the importance of hemoglobin A1c (HbA1c) as a dyslipidemia indication in Afghani individuals with Type 2 Diabetes, the inquiry correlates HbA1c with serum lipid profile. In addition to providing a trustworthy glycemic index, HbA1c may be utilized as an indirect predictor of dyslipidemia [22]. As a result, early detection of dyslipidemia in T2DM patients can help delay the onset of CVD. Diabetes frequently coexists with dyslipidemia that is not recognized. Examining the therapeutic use, the aim of the study is to evaluate lipid profiles and lipid ratios as predictive biochemical models of glycemic control in individuals experiencing type 2 diabetes mellitus (T2DM). TC/HDL-C and LDL-C/HDL-C lipid ratios, as well as lipid profiles (LDL-C) are prospective indicators that may be utilized to forecast glycemic management in T2DM patients [23].

## Conclusion:

Low serum adiponectin and high glycosylation haemoglobin levels association was negative correlation & highly significantly and adiponectin may be a useful marker for risk of T2DM prediction. Exploring interventions to modulate Adiponectin levels for observing their impact on glycemic control could provide valuable strategies for diabetes management and physicians should take this relationship into consideration when applying therapeutic applications. Thus, a diet high in phytates influences adiponectin and HbA1c levels favorably and may help to prevent or minimize diabetic-related complications.

## Ethics approval and consent to participate:

The study was approved by the Institute Ethics Committee, Index Medical College, Hospital & Research Center, Indore, Questionnaire, and informed consent was obtained from all the patients.

## Consent for publication:

All authors have declared that no financial support was received from any organization for the submitted work.

## Funding:

No funding was received for this research.

## List of abbreviations:

T2DM: Type 2 Diabetes Mellitus

HbA1c: glycosylated hemoglobin

EDTA Ethylene-diamine-tetraacetic acid

ADA: American Diabetes Association

COPD: Chronic Obstructive Pulmonary Disease

STD: Sexually Transmitted Diseases

CVD: Cardiovascular Disease

VSMCs: Vascular Smooth Muscle Cells

MCP-1: Monocyte chemoattractant Protein-1

## Figures and Tables

**Table 1:** Baseline characteristics of the patients in each group

**Parameters**	**Diabetes group (n = 105)**	**Control group (n=105)**	**P-value**
Mean age	52.03±10.81	39.13±5.59	0.0001
Sex			
Male	75 (71.42%)	47 (47.00%)	0.0001
Female	30 (28.57%)	53 (53.00%)	
Glycosylated hemoglobin (%)			
Mean value	6.86±0.23	5.57±0.20	0.0001
Adiponectin (ng/ml)			
Mean value	22.71±2.01	8.36±3.17	0.0001

**Table 2:** Depicts the favourable risk factor for identification of type 2 diabetes mellitus

**Test Result Variable(s)**	**AUC**	**95% CI**	**Sensitivity (%)**	**Specificity (%)**	**Best cut-off value**	**Youden Index**
Adiponectin (µg/ml)	0.943	0.942-0.998	99.2	96.8	0.692	0.189
HbA1c (%)	0.91	0.929-0.994	98.1	94.6	0.625	0.161

**Table 3:** Findings of correlation between cases using bivariate correlation

**Parameter**		**Age**	**Adipokines µg/ml**	**HbA1C %**
Age	Pearson Correlation	1	-0.077	-0.115
	Sig. (2-tailed)		0.267	0.098
	N	210	210	210
Adipokines (µg/ml)	Pearson Correlation		1	.675**
	Sig. (2-tailed)			0
	N		210	210
HbA1C (%)	Pearson Correlation			1
	Sig. (2-tailed)			
	N			210
**. Correlation is significant at the 0.01 level (2-tailed).
